# Utilizing geophysical methods for assessing groundwater resources in the Dijil River Catchment, Northwestern Ethiopia

**DOI:** 10.1016/j.heliyon.2024.e38906

**Published:** 2024-10-04

**Authors:** Abatneh Getachew, Yahya Ali Abdulkadir

**Affiliations:** a Amhara Design and Supervision Works Enterprise (ADSWE), Ethiopia; b Department of Physics, University of Gondar, Gondar, Ethiopia

**Keywords:** Groundwater, Vertical electrical sounding, Aquifer characterization, Magnetic method, Dijil river catchment

## Abstract

This study utilizes geophysical methods to assess groundwater resources in the Dijil River catchment near Debremarkos Town, Northwestern Ethiopia. Recent alluvial deposits and volcanic rocks of varying ages characterize the area. The aim is to map subsurface formations and evaluate groundwater potential. Two hundred twenty-eight magnetic data were collected, mainly oriented in NE-SW, NW-SE, E-W, and N-S directions, and twenty-four Vertical Electrical Sounding data utilizing the Schlumberger configuration. The results of the magnetic data reveal lineaments in different directions in the study area. Most of the Geoelectric sections show three layers, of which the second or the third layers are aquifers having minimum and maximum resistivity of 6 and 155 Ohm.m, respectively, and average resistivity range of 13–50 Ohm.m with a thickness ranging 24–200m. The layers represent alluvial deposits, highly weathered and fractured basalt (major aquifer zone), and moderately to slightly weathered and fractured basalt. Shallow and deeper low resistivity horizons, indicating groundwater saturation zones, are visible. The integrated geophysical survey aligns well with available borehole data.

## 1 Introduction

Groundwater investigation in hard rock terrains primarily relies on geophysical techniques to understand groundwater quality and aquifer characteristics and to determine optimal drill locations for efficient resource use [[1], [2], [3], [4], [5], [6], [7], [8]]. Resistivity, electromagnetic, and seismic refraction methods are frequently employed among these techniques.

Geologic structures, such as lineaments and fractures, significantly influence hydrogeological properties in these terrains [9,10]. Wells drilled across these features in structurally controlled areas often exhibit increased productivity, highlighting the importance of mapping subsurface structures. The magnetic method offers a rapid and cost-effective means to achieve this, making it a preferred choice for delineating the fractured basement and studying geological formations [1,[11], [12], [13]].

Among electrical methods, the resistivity survey is a cornerstone of groundwater investigations [14,15]. It enables the identification of lithological contacts and the delineation of water-saturated formations. Notably, the Schlumberger configuration of this method proves particularly useful in locating aquifers for both shallow and deep groundwater exploration [[15], [16], [17], [18], [19]].

**Fig. 1:**
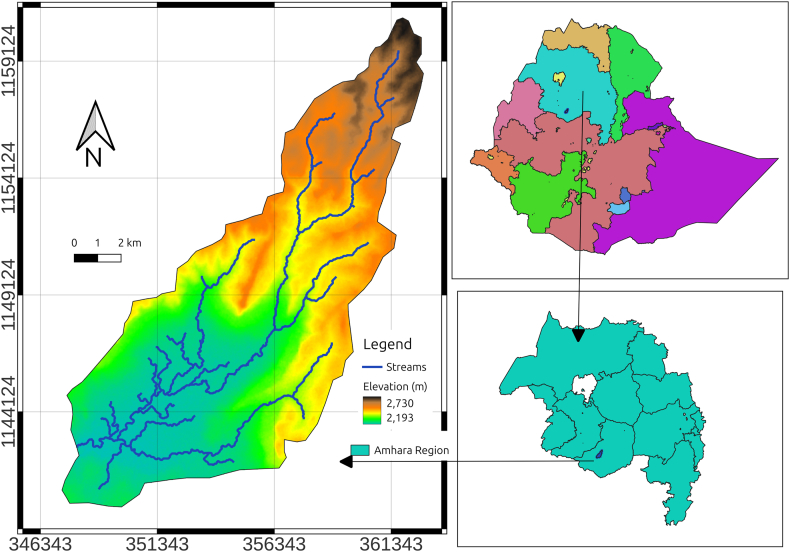
Location map of the study area.

Integrating resistivity and magnetic methods provides a more comprehensive understanding of subsurface hydrology, proving particularly valuable in hard rock environments [1,20,21]. This study utilizes both methods to map subsurface formations and investigate groundwater potential in and around the Dijil catchment in the East Gojjam Administrative Zone near Debremarkos town, Amhara region. The study area is bounded by the UTM-Adindan Coordinate system Zone 37N, with coordinates ranging from (347162–362642) m east and (1139933–1161100) m north (Fig. 1).

## 2 Geology and hydrogeology

**Fig. 2:**
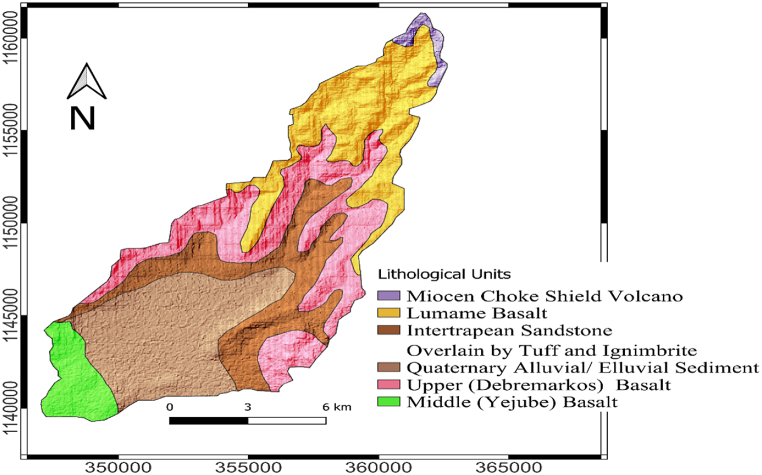
Geologic map of the study area.

A geologic map of the area is presented in Fig. 2. Sandstone units, pyroclastic deposits, Quaternary Alluvial/Elluvial deposits, and basaltic rocks are among the major rock units that comprise the study area's geology and surroundings. The basaltic rock comprises various formations of basaltic rocks and Ignimbrite and tuff deposits. The well-known Choke Shield volcano lies on top of the area. The lowland plain section of the research area is typically covered by Quaternary Elluvial deposits [22,23].

The majority of faults and fractures in the region trend northeast-southwest, east-west, and north-south (Fig. 2). These faults and fractures govern most drainage networks and geomorphological characteristics.

The geologic formations in the area can be divided into two categories: aquifers with unconsolidated sediment and volcanic formations. Alluvial fans along the river and at the foot of the mountains are locations where unconsolidated sediments can be found in the lowland plain of the study area. Alluvial deposits make up this aquifer. Additionally, lava flow is interbedded with thin, locally distributed pyroclastic deposits of varying sizes, facilitating groundwater migration laterally beneath the unconsolidated sediments that comprise the upper aquifer category. However, because of their smaller lateral and vertical extent than the volcanic aquifers, these unconsolidated sediments and pyroclastic deposits are of limited hydrogeological importance in the study area.

Both primary and secondary porosity and permeability are present in volcanic aquifers. The main features of the volcanic aquifers' porosity and permeability are their porous flow textures and vesicles, or gas cavities. Nevertheless, the volcanic aquifers' potential for groundwater is also significantly influenced by weathering and fracturing.

## 3 Methods and materials

This study employed a combined approach of magnetic and electrical resistivity methods to comprehensively understand subsurface structures and groundwater potential. This integration was deemed crucial due to the specific advantages each method offers in the study area's context.

The magnetic method is particularly sensitive to variations in magnetic susceptibility, a physical property that differentiates lithological units and helps delineate geological structures such as faults and fractures [11,[24], [25], [26]]. Identifying these structures was critical in this study as they can act as potential conduits or barriers to groundwater flow in hard rock environments.

The electrical resistivity of rocks is directly related to their lithology and the presence of fluids, making it a valuable tool for groundwater exploration. The Vertical Electrical Sounding (VES) technique was chosen due to its effectiveness in delineating horizontal and sub-horizontal layers typical of sedimentary formations often associated with aquifers. This method is widely recognized for its efficiency in groundwater exploration globally.

### 3.1 Data acquisition

**Fig. 3:**
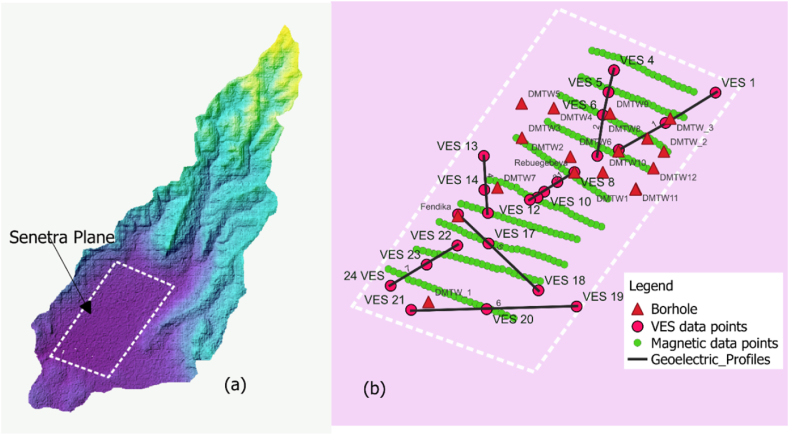
Study area (a) and Distribution of wells and geophysical surveys at Sentera plane.

A GSM-19T type Proton Precision Magnetometer acquired total magnetic field data. Data were collected along ten profiles spanning the survey area, with a station spacing of 100 m, resulting in 228 measurement points (Fig. 3). Twenty-four Vertical Electrical Sounding (VES) data points were collected within the study area (Fig. 3) using an ABEM SAS 4000 Terrameter. The Schlumberger configuration was employed to effectively map both shallow and deep subsurface layers.

### 3.2 Data processing

#### 3.2.1 Magnetic data processing

Reduction to the Equator (RTE) is a crucial process for magnetic data acquired in low-latitude regions. Unlike Reduction to the Pole (RTP), suitable for high latitudes, RTE adjusts magnetic anomalies to simulate data collection under a vertical magnetic field. This reorientation enhances anomaly symmetry and directly indicates subsurface source geometry, improving interpretation accuracy in equatorial zones [27,28]. RTE minimizes the impact of magnetic declination and near-zero inclination, common near the magnetic equator, by effectively centering magnetic anomaly peaks over their corresponding sources [29].

The Tilt Derivative (TDR) technique, particularly useful for delineating shallow basement structures, utilizes both vertical (VDR) and total horizontal (THDR) derivatives of the magnetic field. The TDR equation [insert equation] yields values between -π/2 and +π/2, with zero contours highlighting the edges of geological structures. Positive TDR values generally indicate locations directly above sources, while negative values suggest positions away from them.

Processed magnetic data, including RTE and TDR transformations, contribute to mapping magnetic anomalies. These maps are then interpreted to identify geological boundaries, delineate structures like faults and fractures based on magnetic susceptibility contrasts, and estimate basement depth, ultimately aiding groundwater exploration endeavors.

#### 3.2.2 VES data processing

The apparent resistivity values obtained from VES measurements were plotted on a bi-log graph. These data were then processed using IPI2win software (http://geophys.geol.msu.ru/demo_exe/SetUp_lt.exe). This software employs a one-dimensional (1D) inversion algorithm, which fits a layered earth model to the observed data by minimizing the difference between calculated and measured apparent resistivity values. The inversion process yielded layer parameters for each VES point, including resistivity and thickness/depth.

The interpreted layer parameters from the inversion were then compared to the lithological logs of available boreholes within the study area to correlate resistivity values with specific rock units. This correlation allowed for identifying potential aquifer zones characterized by distinct resistivity ranges.

### 3.3 Data acquisition

A GSM-19T type Proton Precision Magnetometer acquired total magnetic field data. Data were collected along ten profiles spanning the survey area (Fig. 3a), with a station spacing of 100 m, resulting in 228 measurement points (Fig. 3b). Twenty-four Vertical Electrical Sounding (VES) data points were collected within the study area (Fig. 3b) using an ABEM SAS 4000 Terrameter. The Schlumberger configuration was employed to effectively map both shallow and deep subsurface layers.

## 4 Result

### 4.1 Magnetic data interpretation

#### 4.1.1 Total magnetic intensity map

**Fig. 4:**
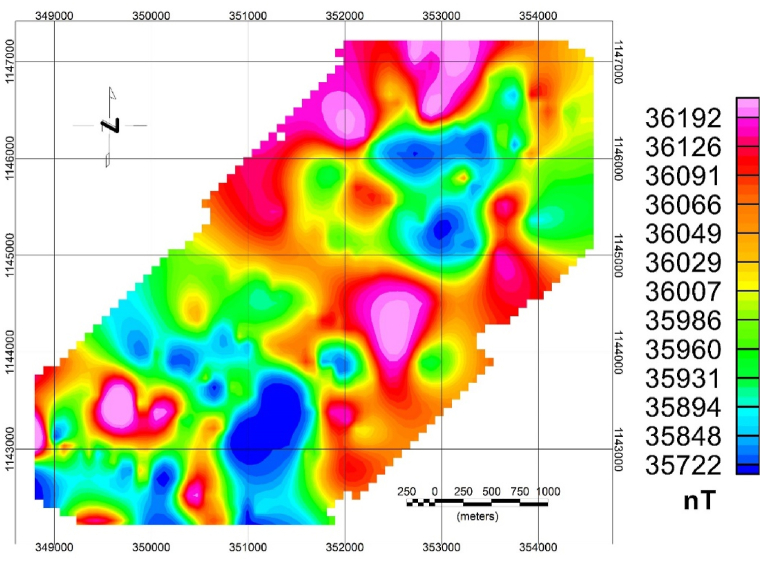
Total magnetic field map of the study area.

Diurnal correction is applied before any processing is applied to the magnetic data. The study area's total magnetic intensity (TMI) anomaly map (Fig. 4) shows the contrast between 35427 and 36501 nT with mean and standard deviation values of 35999 nT and 122.6 nT, respectively. This variation shows contrasting magnetic susceptibilities or variations in structural extends of the rock types in the investigated area.

As shown in the TMI map (Fig. 4), high magnetic anomalies exist in the central and northeastern part of the map while low magnetic anomaly observed on southwestern and some in the NE part of the study area show low magnetic anomaly.

#### 4.1.2 Reduction to equator (RTE) map

**Fig. 5:**
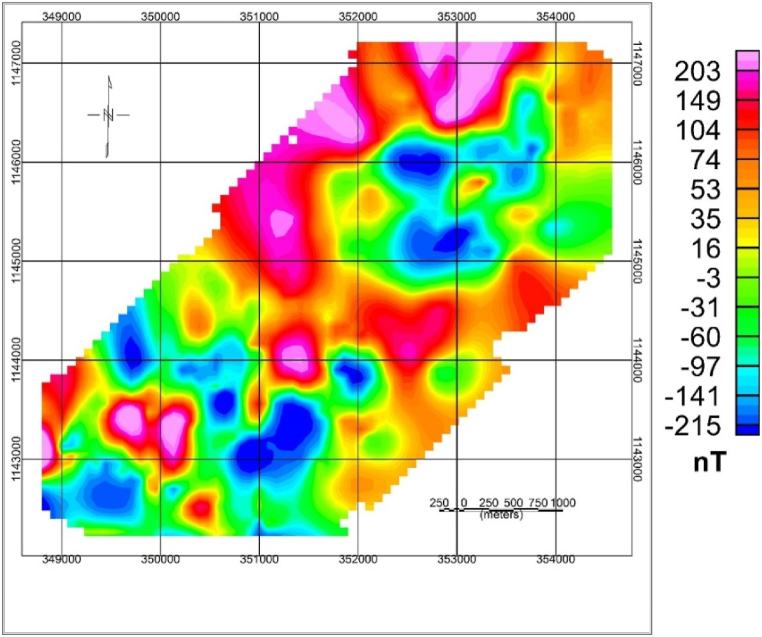
Total magnetic field Reduced to Equator map of study area.

The reduction to the equator map of the study area is presented in Fig. 5. The area's magnetic field inclination and declination are 5.550 and 2.720, respectively, with the main field strength (IGRF) of 35984 nT. The reduction to equator map of the study area is presented in Fig. 5. The area's magnetic field inclination and declination are 5.550 and 2.720, respectively, with the main field strength (IGRF) of 35984 nT.

The area is situated near the magnetic equator, which required removing the influence of declination. The RTE helps remove the magnetic inclination effect in the low magnetic latitude region by centering the peaks of magnetic anomalies over the corresponding sources and helps interpret structural lineaments with their orientations (Olomo et al., 2018).

RTE map is produced after the IGRF value has been removed, as shown in Fig. 5. The map shows the subsurface's overall variation of magnetic responses due to the presence of geological structures and lithological variation. The variation ranges between −370nT and 360 nT.

#### 4.1.3 Tilt derivative (TDR) and lineament maps

The tilt derivative map of the study area is compiled by applying the tilt derivative filter to the RTE magnetic anomaly. The tilt derivative filter is given in equation (Eqn (1)).

(1) $$TDR=\tan-1(\frac{VDR}{THDR})$$

**Fig. 6:**
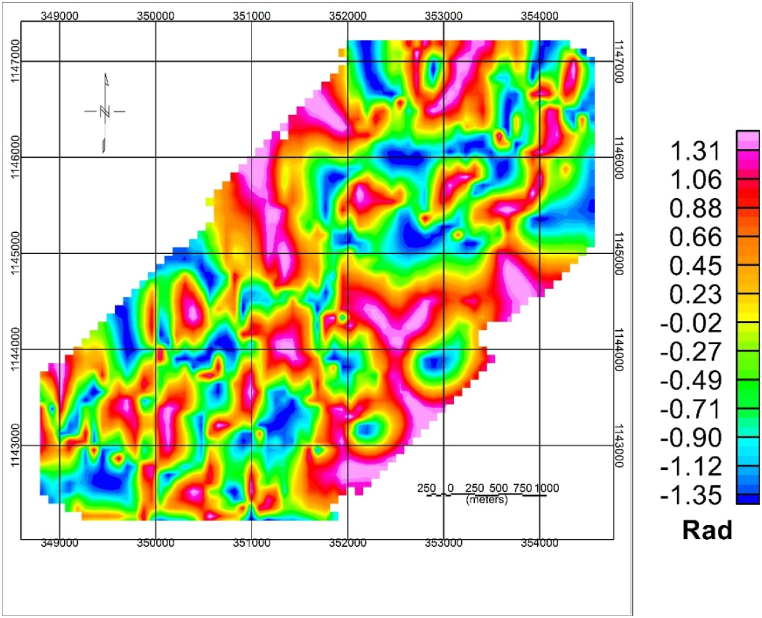
Tilt derivative magnetic anomaly map of the study area.

**Fig. 7:**
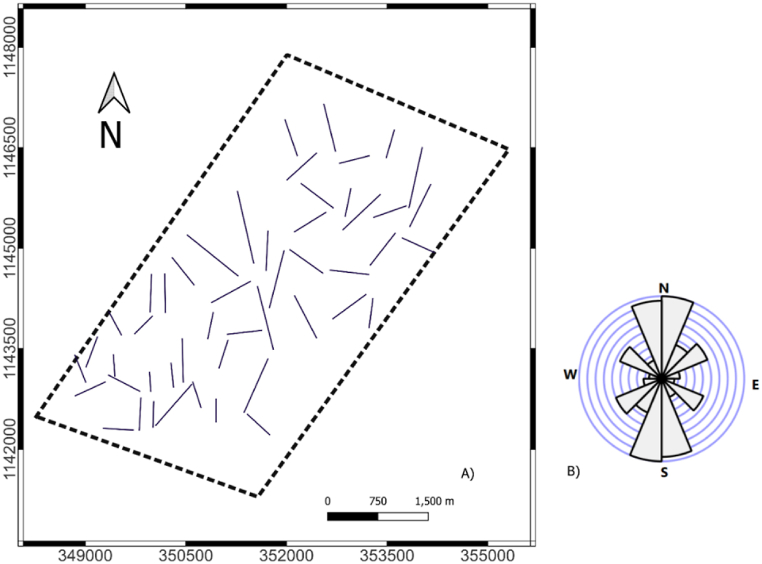
Lineament map of the study area (A), Rose diagram of lineaments (B).

The tilt derivative maps (Fig. 6) show more structural contact/boundary details than the RTE magnetic anomaly map. The zero contour of the tilt derivative is used to extract lineaments. The lineament map is shown in Fig. 7A. A Rose diagram indicating the dominant direction of lineament in the study area is presented in Fig. 7B. The study area shows structural contact/boundary in NW-SE, NE-SW, and N-S direction.

The magnetic lineament map (Fig. 7A) shows the unmapped major magnetic lineament trends in the N to the S direction. Structural high present in the N-S direction of the study area. To identify the distribution of rocks that control the current groundwater flow patterns, tectonics plays a crucial role. The structural highs and lows act as groundwater flow's accumulation and drainage stream channels from a hydrological viewpoint. To obtain accurate subsurface information, geophysical resistivity surveys should generally come after magnetic surveys.

### 4.2 Interpretation of electrical resistivity

Qualitative interpretation of electrical data gives a general view of lateral and vertical variations in the apparent resistivity along the study area.

#### 4.2.1 Sliced-stacked map for different AB/2

**Fig. 8:**
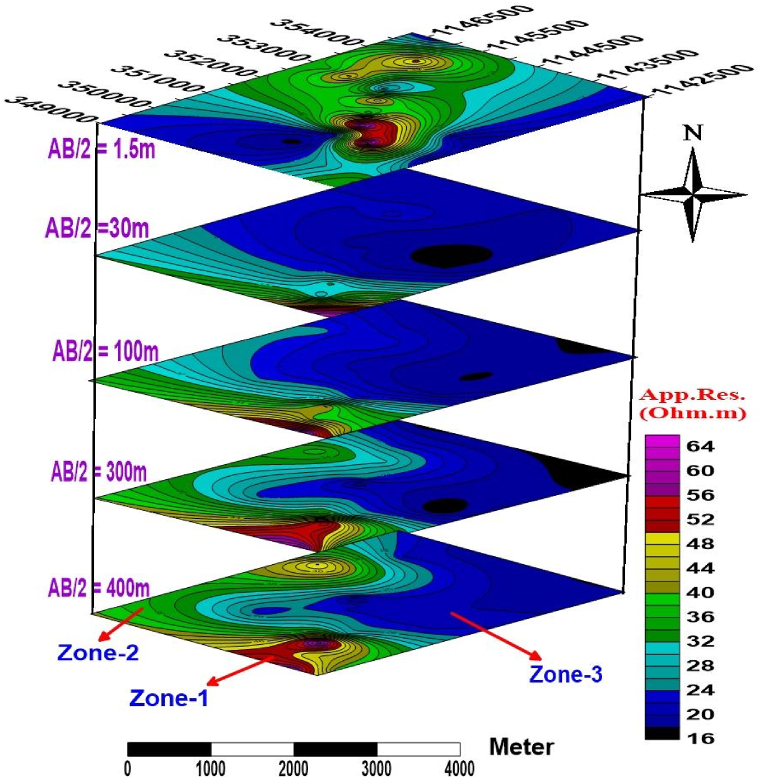
Sliced-Stacked resistivity map.

The total distribution of subsurface geology, both laterally and vertically, in terms of their resistivity is visualized on the sliced stacked map constructed using AB/2 = 1.5, 30, 100, 300, and 400m (Fig. 8). The map shows the relative variation of the apparent resistivity value of the whole area vertically and laterally at different depths of current electrode spacing. Its resistivity value varies from 16 to 64 Ω m.

The study area can be seen as three apparent resistivity zones (**Zone-1, Zone-2, and Zone-3**). Most northern, northeastern, and eastern part of the study area is characterized by low resistivity ranging from 16 to 28 Ω m. On the other hand, a relatively high resistivity value (50–60 Ohm.m) represents the southern and southwestern parts of the area. **Zone**-1 is characterized by relatively high apparent resistivity values, which cover the southern and southwestern part of the study area with a small lateral extension. This high resistivity value is possibly the response of slightly weathered and fractured. **Zone**-2 is characterized by relatively intermediate apparent resistivity values ranging from 36 to 48 Ohm.m, which covers the western, southwestern, and southern part of the study areaand could be the response of moderately weathered and fractured basalt. **Zone**-3 is characterized by a relatively low apparent resistivity value ranging from 16 to 28 Ohm.m. This low resistivity value is possibly the response of water-saturated alluvial deposits at shallow depth and highly weathered and fractured basaltic formation in the deeper part of the survey area. This formation dominates the eastern, northeastern, and southeastern parts. It is found at all depths with variable lateral and vertical extension, and its coverage increases towards the East. It is suitable for groundwater extraction.

#### 4.2.2 Geoelectric sections

**Fig. 9:**
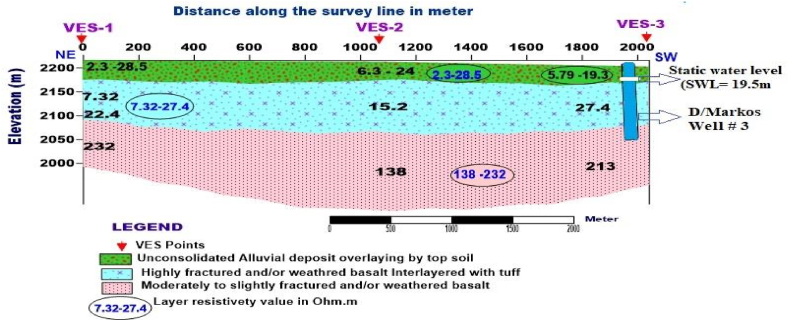
Geoelectric section along profile-1.

**Profile_1:** The geoelectric section for this profile is shown in Fig. 9. The area is characterized by three layers, of which the top layer has variable resistivity ranging from 2.3 to 28.5 Ohm.m and thickness varying from 30.4 to 38.6 m is more likely to be alluvial deposit (a mixture of clay, silty, and sandy soil) overlain by the topsoil as confirmed from the nearby Borehole (Debremarkos well #3, Fig. 16) lithology data near VES-3. The second layer has a relatively low resistivity value ranging from 7.32 to 27.4 Ohm.m and thickness varying 75.4–153m is a response of highly weathered and fractured basalt, the major aquifer zone. The third layer possesses a resistivity value ranging from 138 to 232 Ohm.m, which could be the response of moderately to slightly weathered and fractured basalt.

**Fig. 10:**
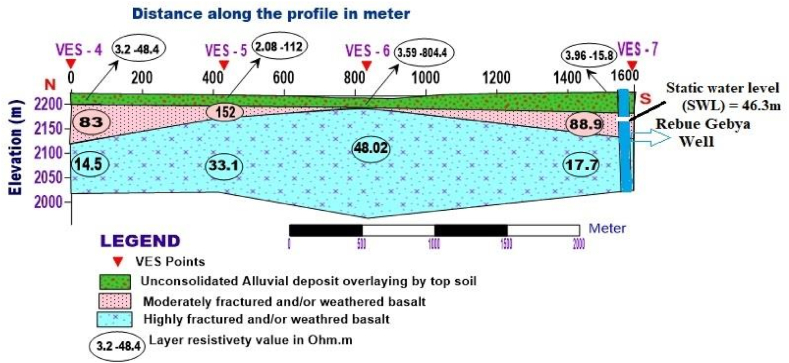
Geoelectric section along profile 2.

**Profile_2:** The Geoelectric section is shown in Fig. 10. This section generally consists of three layers with different resistivity values. The first layer with a resistivity of 2.08–112 Ohm.m and a thickness ranges from 19.83-34m, possibly associated with unconsolidated alluvial deposits, which is composed of clay, silt, and sandy soil overlain by topsoil with different degrees of moisture content. The second layer exhibits relatively medium resistivity values, having a small thickness that could be interpreted as moderately weathered and fractured basalt. The resistivity values of the third layer range from 14.5 to 48.02 Ohm.m, and its depth extends to 24.9–246m around VES 6, which could be a response of highly fractured and weathered basalt, which is the primary aquifer zone compared to from nearby RebuGebeya borehole lithology data located near VES 7.

**Fig. 11:**
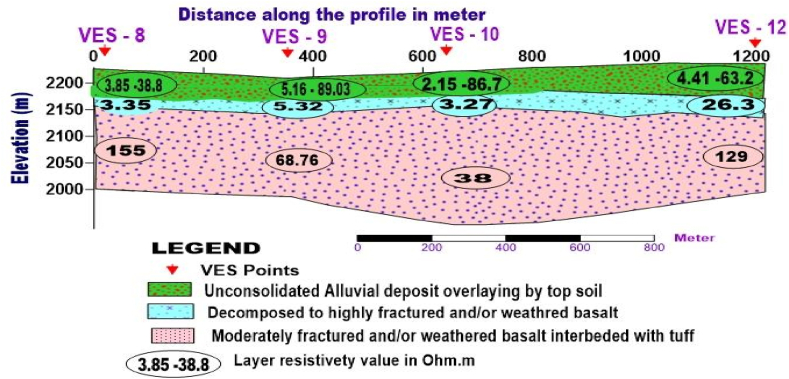
Geoelectric section along profile-3.

**Profile_3:** In Fig. 11, the geoelectric section of profile three is oriented NE to SW direction, representing three distinct layers. The first layer, marked by resistivity values ranging from 3.85 to 89.03 Ohm.m and an average thickness of 30m, responds to unconsolidated alluvial deposit. The second layer, marked by resistivity value 3.27–26.3 Ohm.m with small thickness, is likely correlated with decomposed rock to highly weathered and fractured basalt. The third layer with resistivity values from 38 to 155 Ohm.m and the thickness varying from 59 to 135m is characterized as highly to moderately fractured and weathered basalt.

**Fig. 12:**
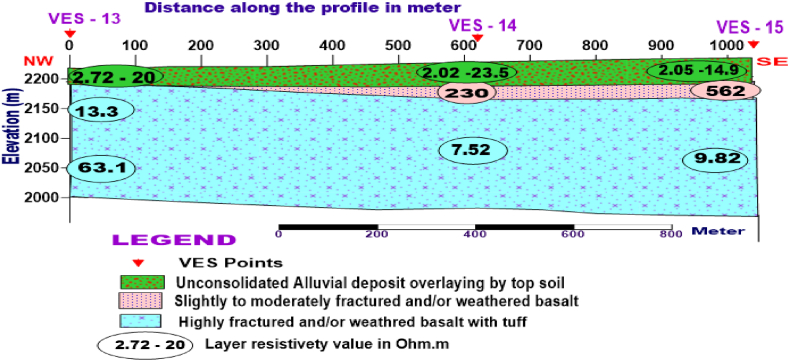
Geoelectric section along profile 4.

**Profile_4:** The Geoelectric section along the profile is constructed, including VES 13, VES 14, and VES 15, which are oriented in the NW- SE direction. Fig. 12 shows three layers with different thicknesses. The top part of the section has resistivity values ranging from 2.02 to 23.5 Ohm.m with thickness varying from 13 to 26.1m is probably related to topsoil mixture of clay, silt, and sand (alluvial deposit). The second thin layer, which does not exist around VES 13 with resistivity varying from 230 to 562 Ohm.m, is geologically interpreted as moderately to slightly fractured and weathered basalt. The third thick layer has relatively low resistivity values ranging from 7.52 to 13. 3Ohm.m mabe the response of the highly weathered and fractured basalt interlayered with tuff.

**Fig. 13:**
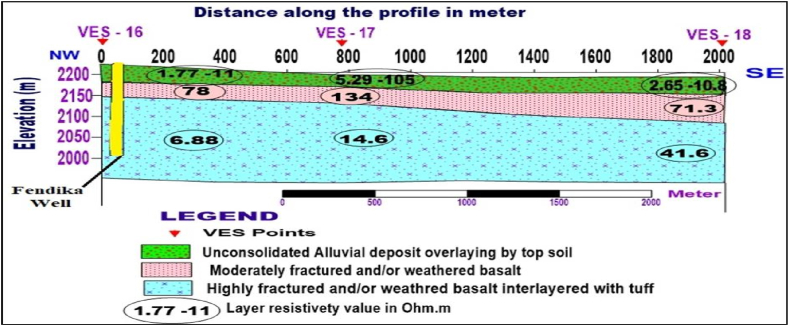
Geoelectric section along profile 5.

**Profile_5:** The Geoelectric section of profile_5 (Fig. 13) was constructed from VES 16, VES 17, and VES 18 along the NW-SE traverse direction. The first geoelectric layer with a resistivity of 1.77–105 Ohm.m, and the thickness varies from 22 to 30m is characterized as topsoil underlain by alluvial deposits with different degrees of saturation. The second geoelectric layer is marked by resistivity values 71.3–134 Ohm.m is likely correlated with moderately weathered and fractured basalt. The resistivity of the third geoelectric layer, which correlated with highly weathered and fractured basalt interlayered with tuff, has a resistivity value of 6.88–41.6 Ohm.m. Because the northwest flank contains a thicker covering of tuff than the southeast, as shown in the section, the Fendika well (Fig. 16) has been abandoned. Therefore, it is usually more practicable to locate groundwater wells in the southeast section of this profile to lessen the likelihood of a dry well.

**Fig. 14:**
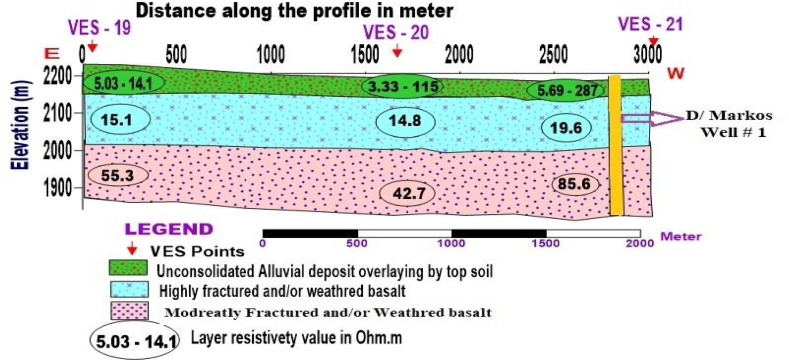
Geoelectric section along profile 6.

**Profile _6:**Fig. 14 presents the Geoelectric section constructed using three VES-sounding data points of VES-19, VES-20 and VES-21. This section was aligned with the E-W direction, and three geoelectrical layers were displayed in. Which the first one with resistivity value varies from 3.33 to 287 Ohm.m is a response of topsoil overlaying alluvial deposits with different degrees of moisture content. The second layer, which is highly fractured and/or weathered basalt, is mapped with resistivity value fluctuating from 14.8 to 19.6 Ohm.m, and total thickness varies 195–258m as referred from the lithological log of Debre Markos well #1 (Fig. 16) close to VES 21. The third layer is interpreted as moderately fractured and/or weathered basalt.

**Fig. 15:**
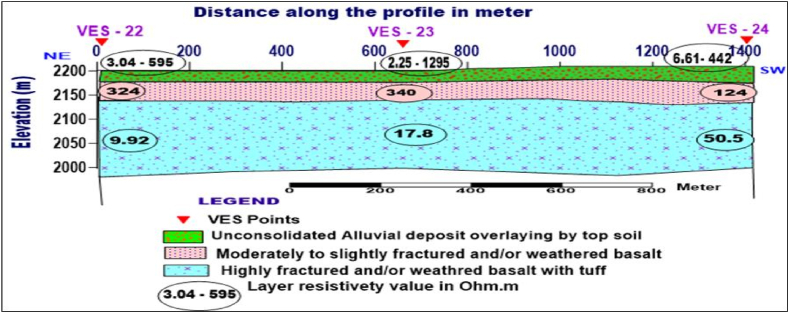
Geoelectric section along profile-7.

**Profile _7:** The Geoelectric section along profile_7 is constructed from VES-22, VES-23, and VES-24, which are oriented in the NE-SW direction (Fig. 15). This geoelectric section was produced to characterize the water-bearing horizon (aquifer zone) near Addise gulit kebele downstream of the study area. Three distinct layers represent the subsurface. The first layer with resistivity value varying from 2.25 to 1295 Ohm.m is the signature of alluvial deposit overlain by top soil with different degrees of moisture content and grain size (clay to bolder). Underplaying the first layer, thin, moderately to slightly fractured and/or weathered basalt with a resistivity value of 124–340 Ohm.m is mapped as the second layer. The third layer, which displays resistivity value between 9.92 and 50.5 Ohm.m, demonstrates highly fractured and/or weathered basalt, the major aquifer zone.

**Fig. 16:**
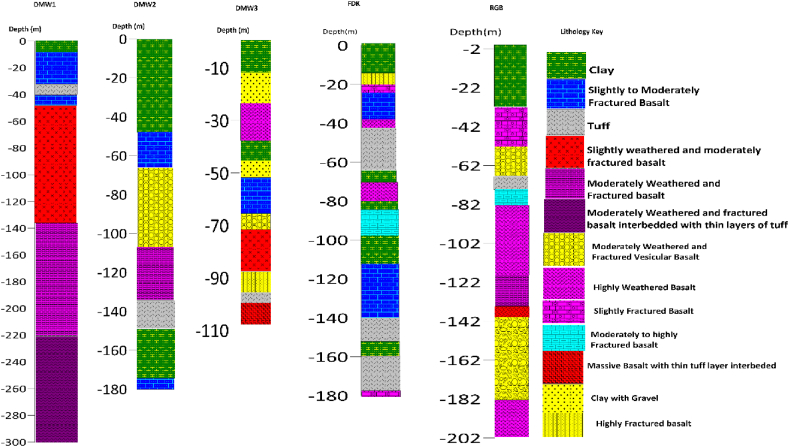
Lithologic description of wells in the study area (DMW-Debremarkos well, FDK-Fendika, RGB-Rebugebeya).

## 5 Discussion

Integrating magnetic and electrical resistivity data provides a powerful approach to delineate subsurface structures and assess groundwater potential in challenging hydrogeological settings like the Dijil River Catchment. The identified NE-SW, E-W, and NW-SE structural trends from the magnetic data are consistent with regional tectonics and likely strongly control groundwater flow [30,31]. These structures may act as preferential pathways for groundwater movement, as evidenced by the spatial correlation between low resistivity zones (interpreted as potential aquifers) and the mapped lineaments [17,32].

The presence of thick, low-resistivity zones at depths ranging from 41.5 m to 273 m, particularly along Profiles 2, 3, 6, and 7, suggests promising locations for groundwater development [5,17,18]. Notably, these zones often coincide with the inferred fault and fracture zones, supporting their role as groundwater recharge and storage conduits. The high-productivity zone identified in the eastern part of the study area aligns with the presence of the Dijil and Wonka Perennial Rivers, suggesting significant surface water-groundwater interaction and potential recharge from these sources.

Previous drilling attempts, such as the abandoned Fendika and Debre Markos well no. 2 and the low-yield Debre Markos well no. 1, likely failed to intersect the identified conductive zones associated with the NE-SW, NW-SE, and N-S trending structures. This highlights the importance of integrating geophysical data for optimal well placement to enhance the success rate of groundwater exploration [33,34].

## 6 Conclusion

This study underscores the effectiveness of integrated geophysical methods, particularly magnetic and electrical resistivity surveys, in deciphering the subsurface hydrogeological framework of the Dijil River Catchment. The results provide compelling evidence for structurally controlled groundwater flow, with NE-SW, E-W, and NW-SE trending lineaments acting as potential groundwater movement and storage conduits. Thick, low-resistivity zones identified at various depths, particularly along the mapped lineaments, represent promising targets for future groundwater exploitation.

The study's findings have important implications for water resource management in the region. The identified potential aquifer zones, particularly those exceeding 300 m in depth, offer opportunities to alleviate water scarcity issues. We recommend drilling along these zones, targeting the intersections with the mapped lineaments to maximize well yields. However, further hydrogeological investigations, including pumping tests and water quality analysis, are crucial to validate these findings and ensure sustainable groundwater management.

## Data availability statement

Data will be made available on request.

## Funding

This research received no external funding.

## CRediT authorship contribution statement

**Abatneh Getachew:** Writing – review & editing, Writing – original draft, Visualization, Software, Methodology, Investigation, Formal analysis. **Yahya Ali Abdulkadir:** Writing – review & editing, Writing – original draft, Methodology, Formal analysis, Conceptualization.

## Declaration of competing interest

The authors declare that they have no known competing financial interests or personal relationships that could have appeared to influence the work reported in this paper.
